# Radar Interferometry Using gNB Base Stations: Estimation and Compensation of Mast Motion and Atmospheric Effects

**DOI:** 10.3390/s26010151

**Published:** 2025-12-25

**Authors:** Alessandra Beni, Lapo Miccinesi, Andrea Cioncolini, Luca Bigazzi, Lorenzo Pagnini, Massimiliano Pieraccini, Sergi Duque, Bleron Klaiqi

**Affiliations:** 1 Department of Information Engineering (DINFO), Università degli Studi di Firenze, 50139 Firenze, Italy; alessandra.beni@unifi.it (A.B.); lapo.miccinesi@unifi.it (L.M.); andrea.cioncolini@unifi.it (A.C.); luca.bigazzi@unifi.it (L.B.); lorenzo.pagnini@unifi.it (L.P.); 2 Huawei Technologies Duesseldorf GmbH, 40549 Düsseldorf, Germany; sergio.duque.biarge@huawei.com; 3 Huawei, 16494 Stockholm, Sweden; bleron.klaiqi@huawei.com

**Keywords:** ground-based radar interferometry (GBRI), structural health monitoring (SHM), joint communication and sensing (JCAS)

## Abstract

**Highlights:**

**What are the main findings?**

- A gNB 5G base station can be effectively used as a ground-based radar interferometer for monitoring structural displacements.
- A regression-based compensation method is proposed to estimate and remove antenna mast motion and atmospheric disturbances directly from radar data, achieving better performance than approaches relying on auxiliary sensors.

**What are the implications of the main findings?**

- Using existing 5G infrastructures for radar sensing significantly reduces deployment and maintenance costs for large-scale Structural Health Monitoring.
- Radar interferometry can be used even for health monitoring of a gNB telecommunication mast itself.

**Abstract:**

Radar interferometry can provide important information for Structural Health Monitoring (SHM) of bridges and other transportation structures. In this article, joint communication and sensing (JCAS) telecommunication infrastructure is tested as a ground-based radar, offering advantages in terms of long-term costs, deployment and maintenance. This work specifically addresses the estimation of the radar support movement (i.e., pylon or mast), which represents a major challenge in this kind of measurements. Movements of the radar system combine with the true target motion and, if not correctly compensated, can compromise the accuracy of the results. A technique for estimating radar movements based on the displacement tracking of multiple permanent scatterers (PSs) in the scenario is presented. True target displacements can then be retrieved by applying linear regression methods to fixed PSs located at different viewing angles, accounting for both radar movements and atmospheric displacement components. The technique was validated using real data acquired during an experimental campaign on a bridge test site. First, results obtained for a target subject to known displacements are shown. A second measurement session was aimed at testing the method for bridge dynamic monitoring. Finally, the same technique was applied antenna mast monitoring in terms of modal analysis and vibration characterization.

## 1. Introduction

Structural health monitoring (SHM) [1,2] is a critical issue for the preservation and safety assessment of bridges and transportation infrastructures. Materials degradation caused by atmospheric agents, combined with continuous usage and heavy loads, can accelerate deterioration processes. Regular maintenance is therefore essential to prevent severe structural damage. A variety of SHM techniques have been proposed in recent years. One common approach relies on the deployment of numerous sensors installed directly on the structure. Fiber optic sensors (FOSs) [3,4] emerged as a highly effective solution due to their inherent advantages, including small size, light weight, and strong anti-electromagnetic interference capability. Other monitoring systems make use of strain gauges [5,6] or accelerometers [7,8]. However, fixed distributed sensor network may be costly to install and maintain, making them unsuitable for continuous and large-scale monitoring. Image-based and computer-vision methods have been introduced as low-cost alternatives for detecting cracks, peeling, deformation, and rusting [9,10,11]. Another efficient, remote sensing technology is radar interferometry. Satellite synthetic aperture radar (SAR) data have proven to be an effective solution for low-cost and large scale monitoring applications [12,13,14,15,16]. However, satellite SAR systems [17] present inherent limitations, particularly in terms of revisit time and illumination geometry.

Ground-based radar interferometers (GBRIs) represent flexible and efficient remote sensing solutions for structural monitoring applications. Owing to their short measurement intervals, typically of the order of milliseconds, GBRIs are particularly suitable for dynamic monitoring tasks. They have been successfully employed for vibration analysis and for measuring slow structural displacements [18,19,20,21,22]. However, GBRIs generally require installation in close proximity to the structure under investigation. Fixed installations can be expensive and, as a result, large-scale or widespread early-warning monitoring remains difficult to implement.

To address challenges related to deployment costs, location constraints, and maintenance, joint communications and sensing (JCAS) infrastructures have recently attracted increasing attention [23,24,25,26,27,28]. Despite significant research interest, JCAS has not yet been widely adopted in operational scenarios, and most existing studies remain at the feasibility or proof-of-concept stage. For example in [26,28], 6G JCAS imaging applications have been studied in controlled environments. More recently, both academic and industrial efforts have explored the use of communication infrastructures for SHM, effectively exploiting them as GBRIs [29]. Leveraging existing, spatially distributed communication infrastructures for monitoring purposes offers substantial advantages in terms of cost reduction and sensor coverage, enabling dense and continuous remote sensing networks. Nevertheless, JCAS-based measurements suffer from intrinsic limitations, including the presence of static and dynamic clutter as well as motion of the radar support (e.g., pylons or masts). These issues have been recently addressed. For instance, in [29,30], the authors proposed a method to remove static and dynamic clutter from a JCAS dataset caused by vehicular traffic, and address the compensation of phase contributions caused by antenna motion.

This work further explores the feasibility of using communication infrastructures as GBRIs, by specifically addressing the estimation and compensation of radar support motion. This issue represents one of the main challenges in JCAS-based measurements, since the radar motion mix with the real movements of the target and, if not compensated correctly, can severely degrade measurement accuracy. In practical scenarios, mast movements, primarily induced by wind and thermal expansion, can reach amplitudes of several tens of centimetres, whereas the structural displacements of interest are typically of the order of a few millimeters. Moreover, the natural frequency of the mast is often comparable to that of the monitored structure, further complicating the separation of the two contributions. Accurate estimation and compensation of radar support motion are therefore essential for reliable displacement measurements.

Alternative solutions based on auxiliary sensors, such as accelerometers, can be adopted to estimate mast motion [31]. However, the integration of additional sensors increases complexity and overall costs. Moreover, the displacement measured by auxiliary sensors must be projected onto the radar line of sight, requiring additional processing steps and potentially introducing further sources of uncertainty.

In this article, a technique for estimating radar support motion based exclusively on radar data is proposed. The method exploits interferometric displacement tracking of multiple stable scatterers in the observed scene, commonly referred to as permanent scatterers (PSs), i.e., targets characterized by high signal quality and negligible motion during the observation interval [32]. Relying solely on radar measurements, the proposed approach does not increase system costs or hardware complexity. The technique is based on linear regression methods and employs a phase model that jointly accounts for both atmospheric effects and mast motion. For the atmospheric contribution, a linear model in the range direction is considered [33]. By exploiting PSs located at different viewing angles, the proposed method enables the estimation of the three orthogonal displacement components of the antenna mast, along with the parameters describing the atmospheric model. These estimates are then used to compensate for the corresponding disturbances and to retrieve the true displacement of the monitored targets.

To the best of the authors’ knowledge, this work presents a novel radar-only approach for estimating and compensating antenna mast motion in JCAS-based interferometric measurements, without relying on auxiliary sensors or external instrumentation. Joint estimation of antenna motion and atmospheric effects results in improved robustness compared to approaches that address these contributions separately. Beyond motion compensation, the estimated three-dimensional mast dynamics are further exploited for modal and spectral analysis, enabling structural health monitoring of the radar support itself. Indeed, knowledge of the mast’s natural oscillation axes and natural frequencies enables modal analysis for early-warning SHM applications [34,35], as variations in these modal parameters can be indicative of changes in the structural characteristics of the mast.

The proposed technique is validated using real data acquired during an experimental campaign conducted in an urban scenario. The dataset was collected by Huawei using a C-band gNB. Results of this study extend the applicability of JCAS infrastructures beyond feasibility studies, demonstrating their potential for reliable displacement monitoring and infrastructure health assessment in realistic urban scenarios.

## 2. Materials and Methods

The proposed method aims to estimate and compensate for the displacement of the antenna support structure by means of a multiparametric regression that jointly models the mast motion and the atmospheric contributions.

For clarity, the problem is first introduced and formalized from a theoretical perspective. Specifically, Section 2.1 presents the mathematical formulation of the proposed compensation method, while Section 2.2 describes the sensing setup and the experimental test site.

### 2.1. Methods Description

**Figure 1:**
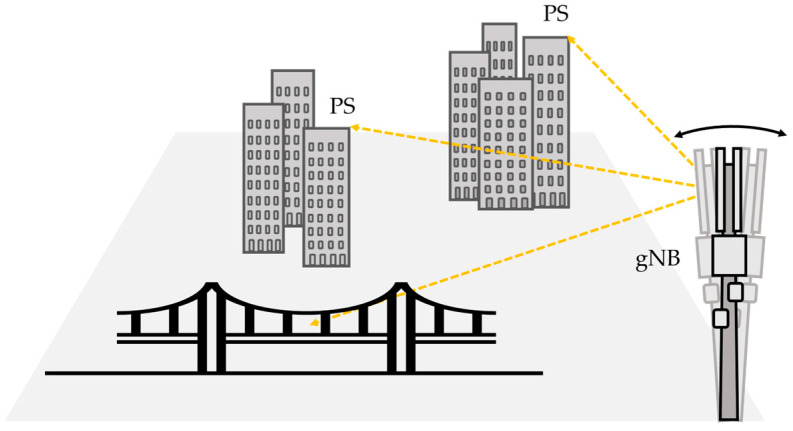
Schematic view of gNB used for JCAS.

JCAS systems are typically based on the next-generation Node B (gNB) antennas, which are commonly installed on pylons (see Figure 1). Motion of the supporting mast can affect the accuracy of displacement measurements of the structure under test.

A radar trace is a complex vector I, whose amplitude provides information on the target location, while the phase can be exploited to detect motion. In particular, target displacement can be retrieved through interferometric processing. The interferometric phase, i.e., the phase difference between subsequent acquisitions, of a target acquired with a gNB can be modelled as a sum of four terms:

(1) $$\Delta \varphi =\Delta \varphi_{disp}+\Delta \varphi_{ant}+\Delta \varphi_{atmo}+\Delta \varphi_{calib},$$

where $\Delta \varphi_{disp}=4\pi /\lambda \cdot \Delta R$ is the term due to the physical displacement, ΔR, of the target, and λ the wavelength; $\Delta \varphi_{ant}$ is the phase contribution due to the mast movement; $\Delta \varphi_{atmo}$ is the atmospheric term, given by a variation in the air refractive index between different acquisitions; and $\Delta \varphi_{calib}$ is a calibration term due to the hardware characteristics. In most of the cases the removal of $\Delta \varphi_{calib}$ is trivial since it can be modelled as a constant phase that does not depend on the selected target. In what follows, $\Delta \varphi_{calib}$ is not considered.

The proposed method aims to isolate the displacement term $\Delta \varphi_{disp}$, through estimation and compensation of all the other terms.

Compensation of the antennas and atmospheric phase contributions can be implemented exploiting information from PSs, pixels that remain coherent throughout the entire observation interval [36]. These pixels can be identified through the analysis of the time series of their amplitude values, with the evaluation of the amplitude dispersion index ($D_{A}$) [32,37]. PSs are selected by considering only those pixels with $D_{A}$ below a given threshold, typically $D_{A\;}$ < 0.25.

The atmospheric phase term arises from changes in the air’s refractive index between acquisitions [38]. These changes in atmospheric refractivity induce propagation delays in the radar signal, which depend on the path between the radar to the target, i.e., the range distance. Under the assumption of uniform atmospheric parameters, which is usually assumed for flat terrain, this contribution exhibits a linear dependence on range [33]. The atmospheric term can be estimated by assuming a linear range dependence of the phase term, using multiple linear regression based on the phase values of the PSs [39,40].

**Figure 2:**
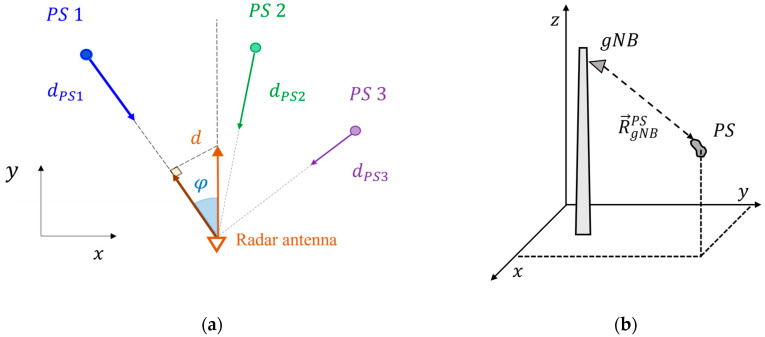
Scheme of the geometry of the proposed method: (**a**) 2D view; (**b**) 3D view.

With regard to the antenna term, several considerations are required. This term is proportional to the projection of the antenna displacement vector on the radar line-of-sight. Therefore, it depends on the physical position of each target, as schematically illustrated in Figure 2a. As an example, consider a PS located at an angle φ with respect to the gNB orientation. If the gNB undergoes a displacement d along the y-axis while the PS remains motionless, the measured interferometric displacement of that PS, denoted as $d_{PS}$, corresponds to the projection of the antenna displacement along the radar line of sight, i.e., $d_{PS}=d\;cos(\varphi )$.

For general antenna movements of components ($d_{x}^{ant},d_{y}^{ant},d_{z}^{ant}$), the PS measured displacement can be expressed as:

(2) $$d_{PS}=M_{x}^{PS}d_{x}^{ant}+M_{y}^{PS}d_{y}^{ant}+M_{z}^{PS}d_{z}^{ant},$$

where $M_{x}^{PS},M_{y}^{PS},M_{z}^{PS}$ are the projection coefficients of the cartesian displacement components along the gNB-target direction, determined by the selected PS positions:

(3) $$M_{i}^{PS}=\frac{{\vec{R}}_{gNB}^{PS}\cdot {\hat{e}}_{i}}{\left|{\vec{R}}_{gNB}^{PS}\right|},\;i=x,y,z,$$

where ${\vec{R}}_{gNB}^{PS}$ is the gNB-target vector (Figure 2b), and ${\hat{e}}_{i},\;i=x,y,z$ is the unit vector of the coordinate axis i.

Therefore, for a specific target, the phase contribution due to the antenna displacement can be written in terms of the orthogonal components of the antenna displacement ($d_{x}^{ant},d_{y}^{ant},d_{z}^{ant}$) as

(4) $$\Delta \varphi_{ant}=\frac{4\pi }{\lambda }(M_{x}^{PS}d_{x}^{ant}+M_{y}^{PS}d_{y}^{ant}+M_{z}^{PS}d_{z}^{ant}).$$

As mentioned above, the atmospheric contribution can be modelled as a linear relation along range. That is,

(5) $$\Delta \varphi_{atmo}=\varphi_{0}+\varphi_{1}R,$$

where R is the range distance radar–target, $\varphi_{0}$ and $\varphi_{1}$ are constant parameters.

Then, for each PS the phase difference can be written as

(6) $$\Delta \varphi_{PS_{i}}=\frac{4\pi }{\lambda }\left(M_{x}^{PS_{i}}d_{x}^{ant}+M_{y}^{PS_{i}}d_{y}^{ant}+M_{z}^{PS_{i}}d_{z}^{ant}\right)+\varphi_{0}+R^{PS_{i}}\varphi_{1}.$$

Here, $d_{x}^{ant},d_{y}^{ant},d_{z}^{ant},\varphi_{1},\varphi_{0}$ are the five unknown parameters to be estimated. Joint estimation of antenna motion and atmospheric effects leads to a more robust estimation than analysing them independently.

The phase model (6) can be written for all the PSs and for each interferometric couple, as a linear system using the matrix form:

(7) $$\Delta \varphi_{PS}=Ax.$$

Here, $\Delta \varphi_{PS}={\left(\Delta \varphi_{PS}^{1},\Delta \varphi_{PS}^{2},\;\ldots ,\;\Delta \varphi_{PS}^{N}\right)}^{T}$ is the vector of the phase difference measured for all the N PS; and the matrix A is defined as

(8) $$A=\left(\begin{array}{ccccc} M_{x}^{1} & M_{y}^{1} & M_{z}^{1} & 1 & R^{1}\\ M_{x}^{2} & M_{y}^{2} & M_{z}^{2} & 1 & R^{2}\\ \ldots & \ldots & \ldots & \ldots & \ldots \\ M_{x}^{N} & M_{y}^{N} & M_{z}^{N} & 1 & R^{N} \end{array}\right),$$

where the apex indicates the i-th PS; and the vector x is defined as

(9) $$x={\left(d_{x}^{ant},d_{y}^{ant},d_{z}^{ant},\varphi_{0},\varphi_{1}\right)}^{T}.$$

A solution of the linear system (7) is given by the Ordinary Least Squares (OLS) solution,

(10) $$\hat{x}={\left(A^{T}A\right)}^{-1}A^{T}\Delta \varphi_{PS}.$$

From the estimated parameters ($\hat{x}$), it is possible to retrieve the estimated phase disturbances, i.e., antenna mast movement and atmospheric contribution, of any target in the scene located in position (x,y,z) at time t with:

(11) $$\Delta \hat{\varphi }\left(x,y,z,t\right)=\left(\begin{array}{ccccc} M_{x}^{target} & M_{y}^{target} & M_{z}^{target} & 1 & R^{target} \end{array}\right)\hat{x};$$

where $M_{j}^{target}$, j=x,y,z are the projection coefficients of the target, and $R^{target}$ is the radar–target distance.

To compensate for mast movements and atmosphere effects, the cumulative phase ($\Delta {\hat{\varphi }}_{cum})$ has to be considered:

(12) $$\Delta {\hat{\varphi }}_{cum}(x,y,z,t)=\;\sum_{t^{\prime }=0}^{t^{\prime }=t}\Delta \hat{\varphi }(x,y,z,t^{\prime }).$$

Finally, the image I at time t can be corrected as follows

(13) $$I_{corr}(x,y,z,t)=I\cdot e^{-j\Delta {\hat{\varphi }}_{cum}(x,y,z,t)}.$$

The estimated $I_{corr}$ is no longer affected by disturbance and can be used to calculate the displacement using interferometric processing.

To evaluate the disturbance rejection capability of the proposed method, a spectral analysis can be performed. This analysis provides a quantitative assessment of the effectiveness of disturbance suppression. For structural monitoring applications, the spectral analysis can be implemented using Joint Time–Frequency Analysis (JTFA) [41], which computes the Fourier transform over a sliding time window.

### 2.2. Sensor and Test Site Description

The proposed method was tested using data acquired by Huawei from a C-band gNB in an urban area in Nanjing, China. In general, gNBs exploit orthogonal frequency-division multiplexing (OFDM) signals, in which one or more symbols can be used for radar sensing applications. Moreover, gNBs are equipped with multiple input multiple output (MIMO) antennas, which enable angular resolution capabilities [42]. The MIMO antennas are used for beam steering, whereby the gNB sequentially acquires data at different angles. For the purposes of the present work, acquisitions at different angles can be considered quasi-simultaneous. The pulse repetition frequency (PRF) of the gNB for JCAS applications depends on the system configuration and ranges from a few Hz up to several kHz.

**Figure 3:**
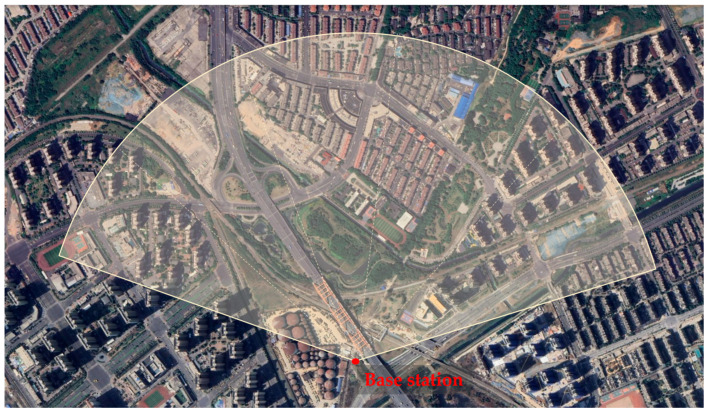
Aerial view of the scenario.

The illuminated scenario is shown in Figure 3. It includes several buildings of different heights, green areas and a bridge, near to the gNB. These buildings were selected as PSs, since they were characterized by a strong and stable signal and are essentially motionless.

**Figure 4:**
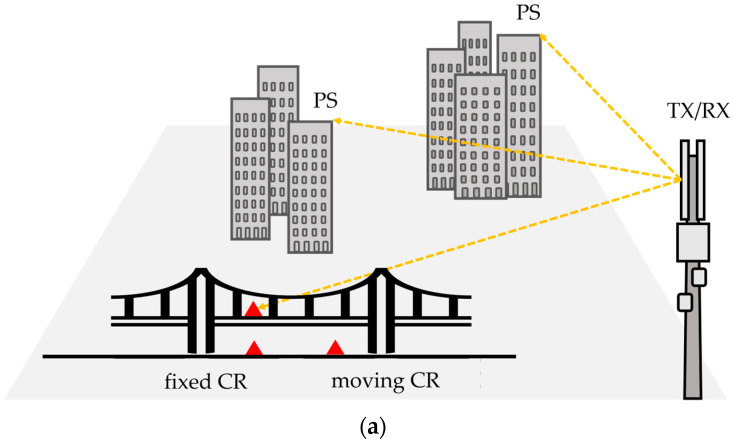

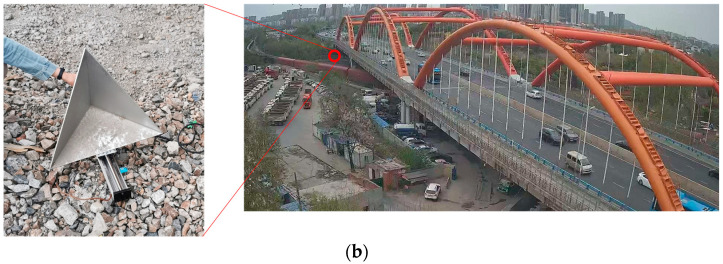
Scheme of the scenario: (**a**) spatial geometry; (**b**) moving CR located on a linear actuator. (red triangles indicate CRs).

Three corner reflectors (CRs) were located in the vicinity of the bridge and served as reference targets. A schematic representation of the environment, including the locations of the CRs, is shown in Figure 4a. One CR was placed on the bridge deck to measure the bridge displacement. It was positioned at 236 m from the gNB, at an azimuth angle 17° and an elevation of +3.4°. One other CR was located on the ground under the bridge, aligned with the same azimuth angle of the first, but at an elevation of −9° and a distance of 238 m from the gNB. The third CR was placed on a linear actuator on the ground, 207.8 m from the gNB, at an azimuth angle of 18.05° and an elevation of −6.07°, as shown in Figure 4b. This CR was used to simulate controlled displacement for testing the system’s long-term monitoring capabilities.

The gNB mast, typically made of steel, can reach heights of approximately 30 m. Therefore, wind loads and thermal effects can significantly influence its structural behaviour.

## 3. Results

Three measurement sessions are presented in this section. The first session was conducted to retrieve the quasi-static movement of a target; the second aimed to evaluate the performance of the proposed method for dynamic monitoring of a bridge; the third session was designed to assess the capability of gNB interferometry to monitor the mast itself. The experimental scenario is described in Section 2 (see Figure 3). The CR mounted on the small actuator was used for the quasi-static test and removed for the dynamic monitoring experiment. The system acquired data with a PRF of 400 Hz. Dynamic clutter induced by vehicular traffic mainly affected targets located on the bridge deck and on the ground, such as the corner reflectors. To mitigate these effects, noise reduction was performed directly on the complex radar data prior to persistent scatterer (PS) selection. Given that the structural vibrations of interest were typically below 10 Hz, temporal averaging over groups of 40 consecutive acquisitions was applied, effectively reducing the PRF from 400 Hz to 10 Hz while improving the signal-to-noise ratio. Subsequently, a low-pass filtering stage was applied in the 0–10 Hz band using a 9th-order Butterworth digital filter to further suppress high-frequency noise and dynamic clutter components. The coordinates of each PS were retrieved by considering the range and the beam steering angle. This information was mapped in a satellite view and the coordinates in longitudinal-latitude grid were extracted.

The measurements are influenced by both atmospheric effects and antenna mast movements. In particular, mast motion consists of two components, a fast vibration and a slow displacement (commonly referred to as the sway effect [31,43,44]) caused by thermal expansion of the mast.

**Figure 5:**
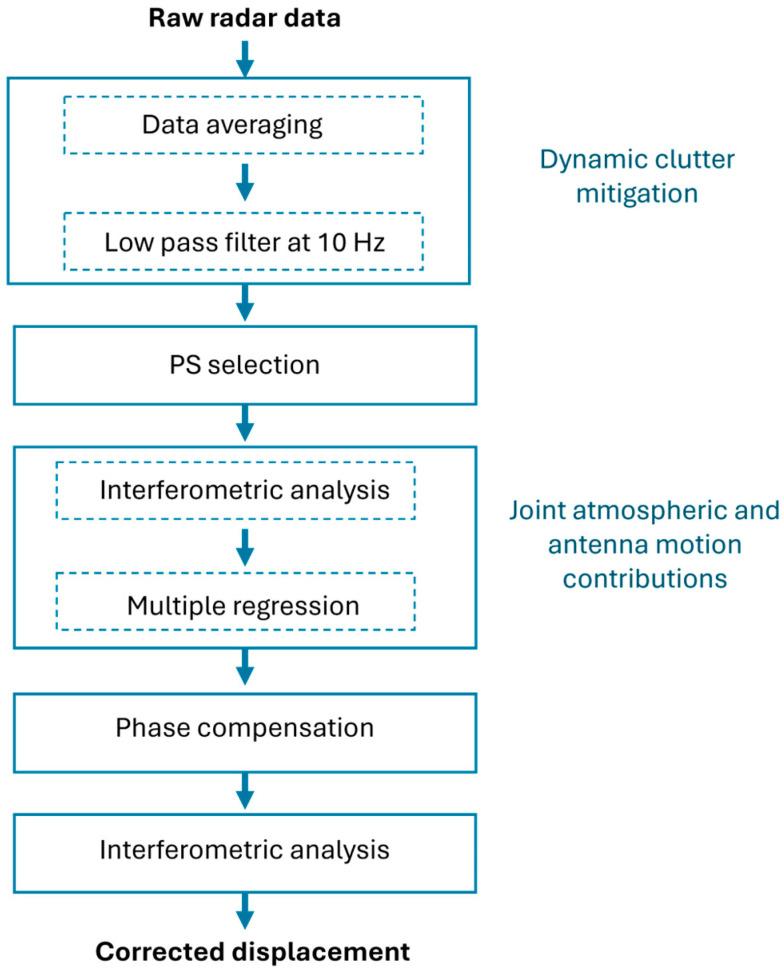
Flow diagram illustrating the processing chain of the proposed technique.

The overall processing chain is summarized in the flow diagram shown in Figure 5. Raw data are first processed to mitigate high-frequency noise and dynamic clutter. Subsequently, PSs are selected based on the amplitude time series. Joint estimation of antenna motion and atmospheric contributions is then performed through interferometric analysis and multiple linear regression, according to Equations (6)–(10) introduced in Section 2.1. Phase compensation is subsequently applied using Equations (11)–(13). Finally, the corrected interferometric displacement of the monitored target is retrieved through interferometric analysis.

### 3.1. Quasi-Static Monitoring

**Figure 6:**
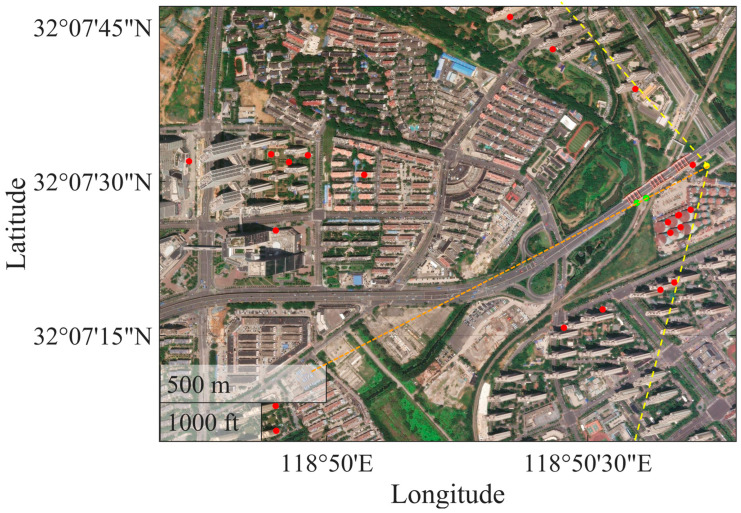
Map of the illuminated area. Red dots correspond to PSs, the green dots to the CRs, while the yellow dot indicates the gNB position.

The duration of the first measurement session was about 12 h. The CR on the actuator was moved step-by-step by 1 mm per hour. The cumulative interferometric displacement of the entire measurement was calculated for each target. The PSs used for this measurement were 23. Figure 6 illustrates the spatial distribution of the PSs over the illuminated area, with red dots indicating the positions of the PSs, green dots representing the corner reflectors, and a yellow dot marking the location of the gNB. The orange dotted line indicates the direction orthogonal to the radar line of sight. The PSs are distributed over an azimuthal range of approximately 126° and an elevation range of 23.7°, from +5.2 to −18.5°.

**Figure 7:**
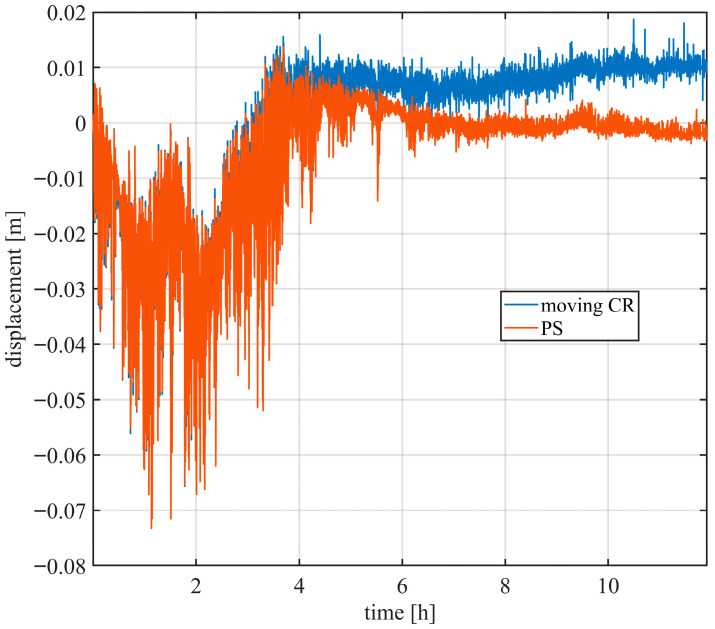
Example of displacement of a PS and moving CR before disturbance removal.

Figure 7 shows the measured displacement of one PS and of the moving CR. Before disturbance compensation, the targets exhibit similar displacement trends: during the first four hours, large displacements of up to 10 cm are observed.

**Figure 8:**
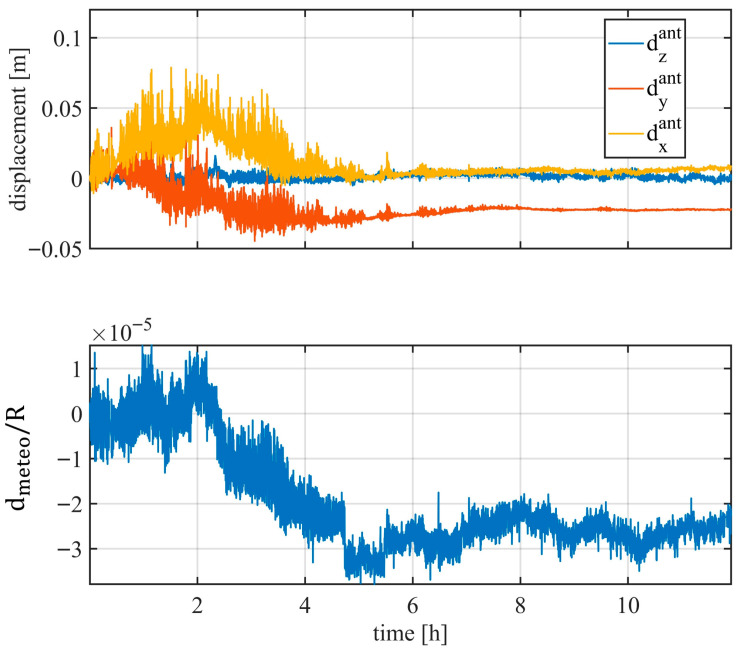
Disturbance coefficient retrieved with (10).

The disturbance was estimated using Equation (10), and the retrieved coefficients are shown in Figure 8. The atmospheric effect in Figure 8 is normalized with respect to the range. It is worth noting that the displacement of PSs during the first four hours is influenced by both mast motion and atmospheric variations. From the fourth hour onward, mast movement becomes negligible, while the atmospheric variations remain small.

**Figure 9:**
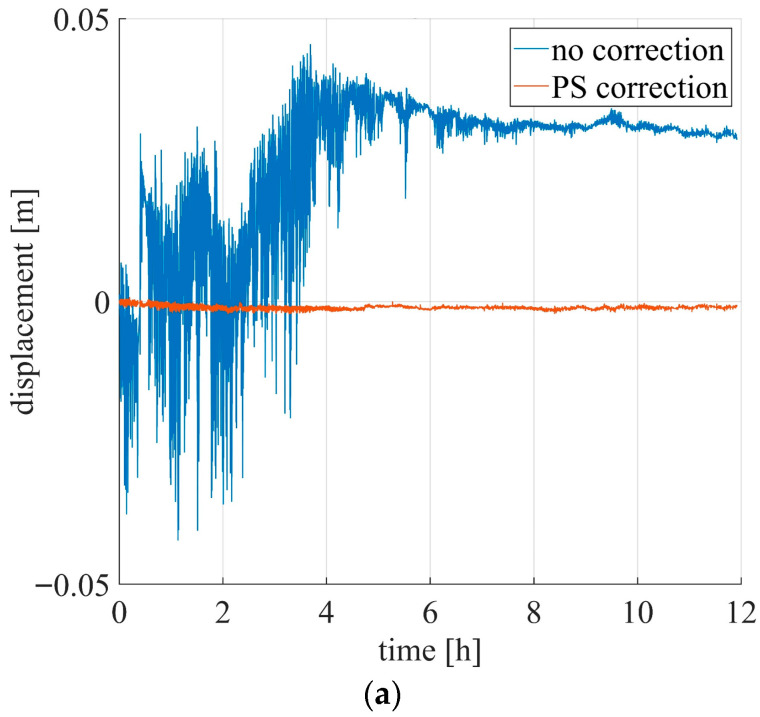

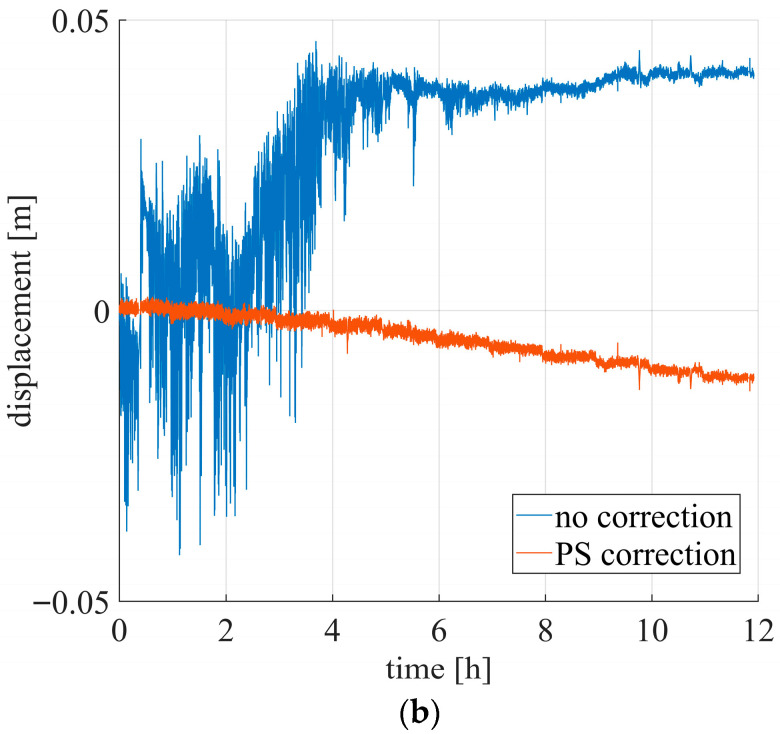
Displacement before (blue) and after (orange) disturbance removal: (**a**) displacement of a PS; (**b**) displacement of the moving CR.

**Figure 10:**
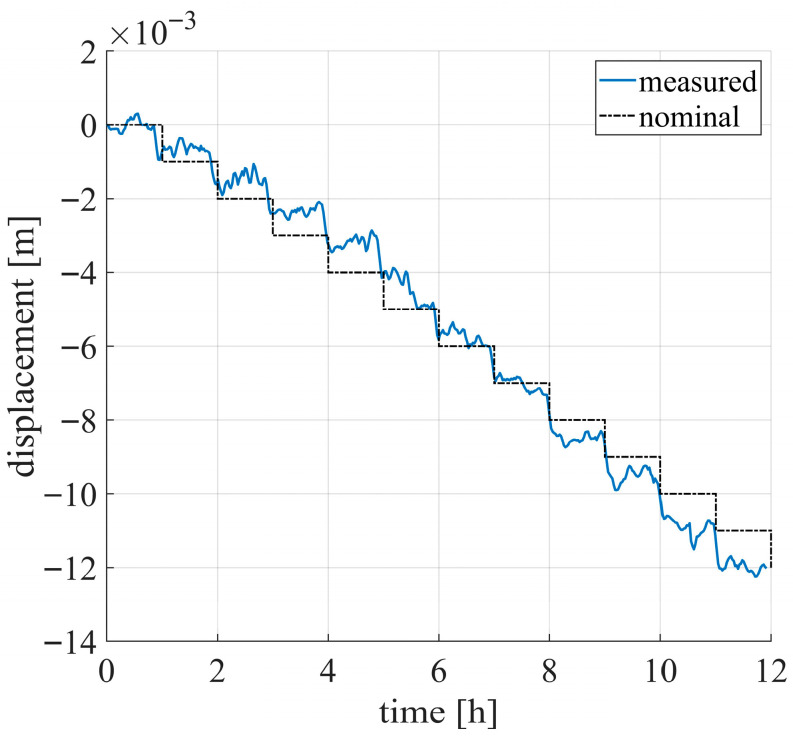
Displacement of moving CR after low-pass filtering.

Figure 9 shows the displacement of the two targets before and after the disturbance compensation. After compensation, as expected, the PS is essentially motionless (orange line in Figure 9a), with a mean displacement of −1.1 mm and a standard deviation of 0.3 mm. The displacement of the CR (orange line in Figure 9b) exhibits a downward trend related to motion of the actuator. To better visualize the CR displacement, a low-pass filter was applied using a moving average with a 300 s time window. The result is shown in Figure 10. It can be observed that a 1 mm step occurs every hour, which is consistent with the nominal actuator displacement (black dotted line in Figure 10).

### 3.2. Dynamic Monitoring Under Vehicular Traffic Stimulus

The second measurement session lasted approximately 10 h. To investigate the dynamic behaviour of the bridge excited by vehicular traffic, the CR installed on the bridge (see Figure 4) was used as the monitored target, with vehicular traffic serving as the excitation.

**Figure 11:**
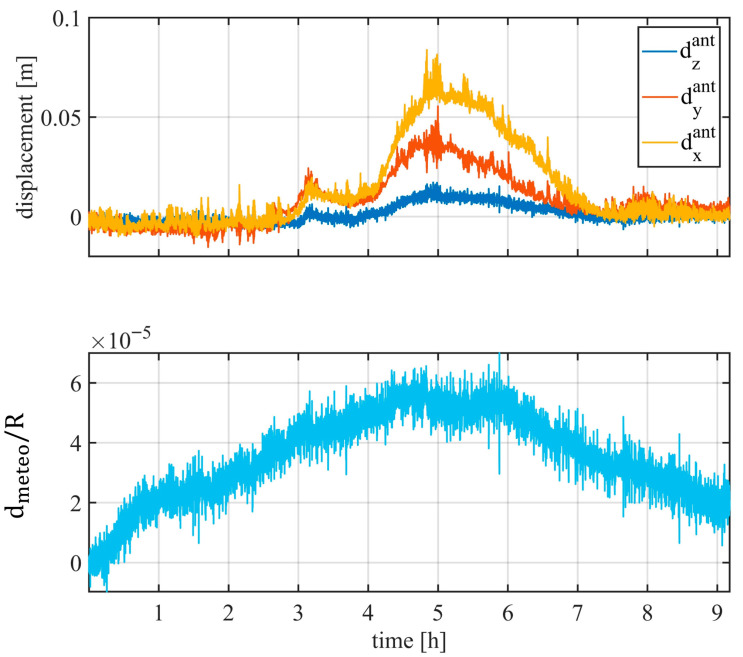
Disturbance coefficient retrieved with (10) (using 23 PSs).

Equation (10) was evaluated using 23 PSs, and the retrieved coefficients are shown in Figure 11. The spatial distribution of the PSs was the same as illustrated in Section 3.1 (Figure 6). In this case as well, significant antenna mast displacements were observed in x and y directions, corresponding to the typical sway effect affecting telecommunication towers.

**Figure 12:**
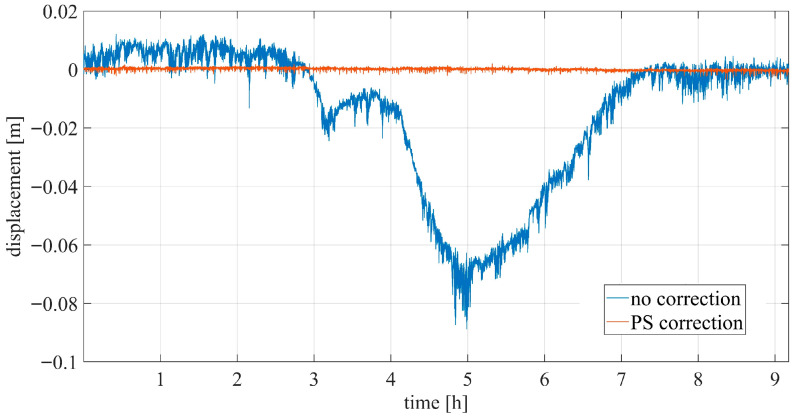
Interferometric displacement of the targets before correction (blue) and after correction (orange).

**Figure 13:**
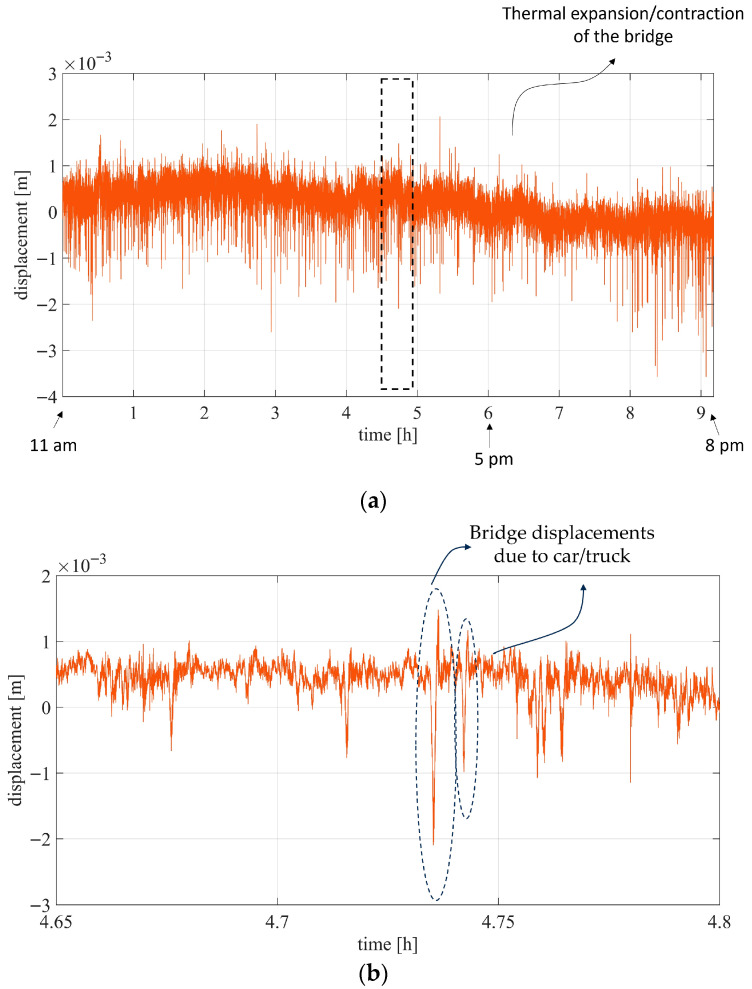
Interferometric displacement of the targets after correction: (**a**) whole measurement duration; (**b**) detail of displacement around 4.65 h and 4.8 h.

**Figure 14:**
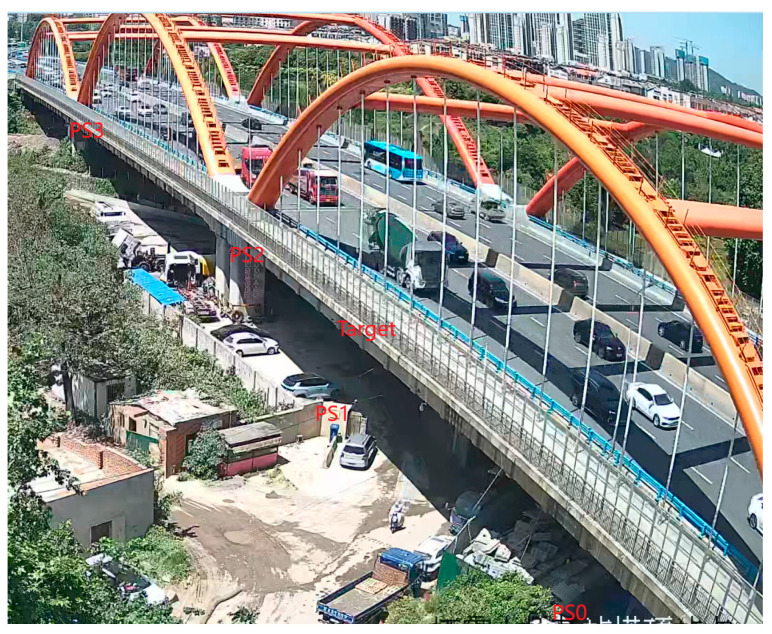
Vehicular traffic on the bridge under test.

Figure 12 shows the displacement of the CR on the bridge before and after correction. After compensation, the CR is almost stable with no evident trend. However, a small residual trend is visible in the corrected displacement (Figure 13a), likely related to thermal expansion or contraction of the bridge, since the measurements were taken between 11 am and 8 pm. A detailed view of the displacement over a specific time interval is shown in Figure 13b, where several large displacement peaks (up to 3.5 mm peak-to-peak) can be observed, corresponding to the excitation due to vehicular traffic (Figure 14).

**Figure 15:**
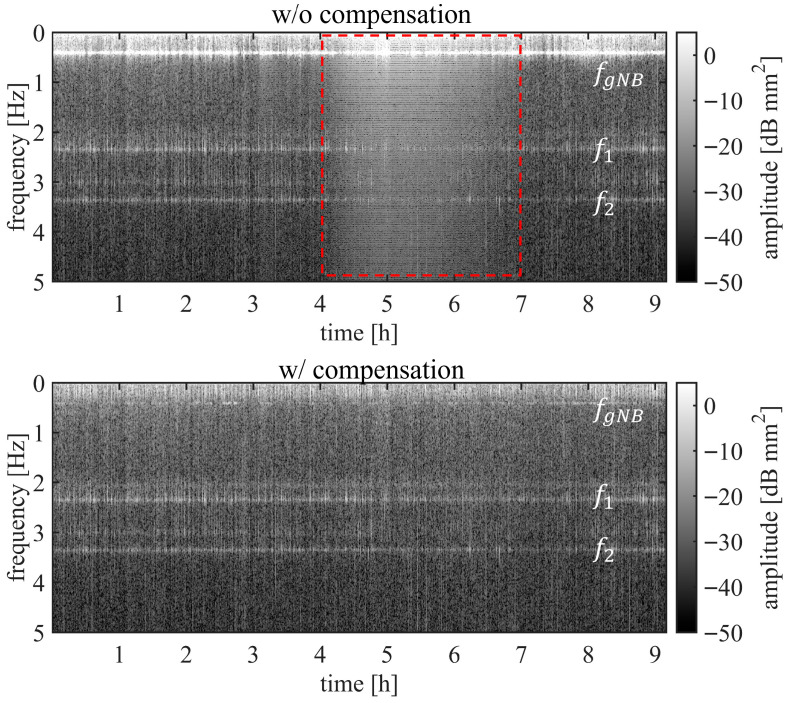
Fourier transform of displacement of Figure 13a calculated using a sliding window of 50 s.

Figure 15 shows the JTFA of the displacement in Figure 12, calculated over a sliding window of 50 s. Three main frequencies are identified: $f_{gNB}=0.40\;Hz$, $f_{1}=2.33\;Hz$, and $f_{2}=3.33\;Hz$. The first frequency ($f_{gNB}$) dominates in the uncompensated data but nearly disappears after compensation, indicating that it is associated with the gNB movement. The proposed method enables the identification of the bridge’s natural frequencies even during the fourth to seventh hour, where the spectrum is otherwise obscured by noise (dotted red square in Figure 15).

**Figure 16:**
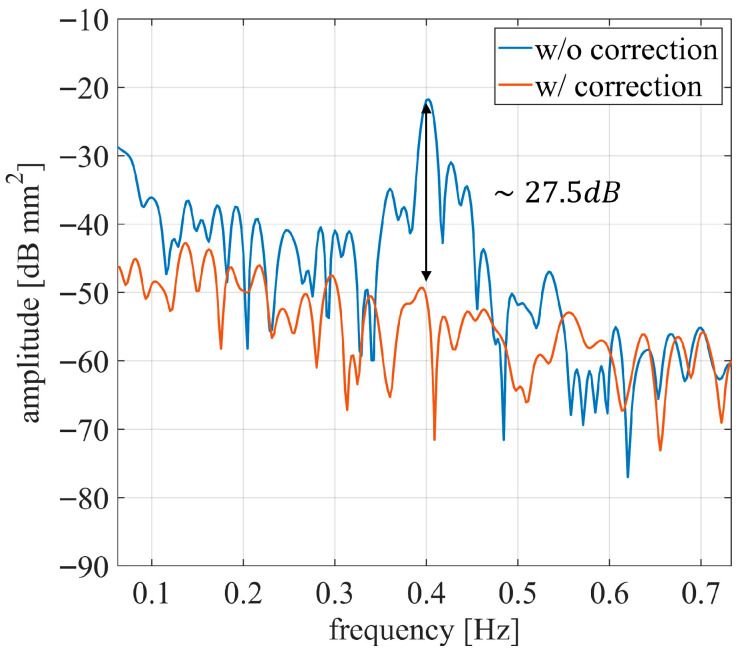
Fourier transform of the two stimuli of Figure 13b.

To quantify disturbance suppression, the Fourier transform of the CR displacement shown in Figure 13b was calculated. Figure 16 presents the results: before compensation, a peak at 0.40 Hz is visible, which corresponds to the main frequency of mast motion. After disturbance suppression, this peak is reduced by approximately 27.5 dB, demonstrating the effectiveness of the proposed method.

### 3.3. Telecommunication Mast Monitoring by Interferometry

**Figure 17:**
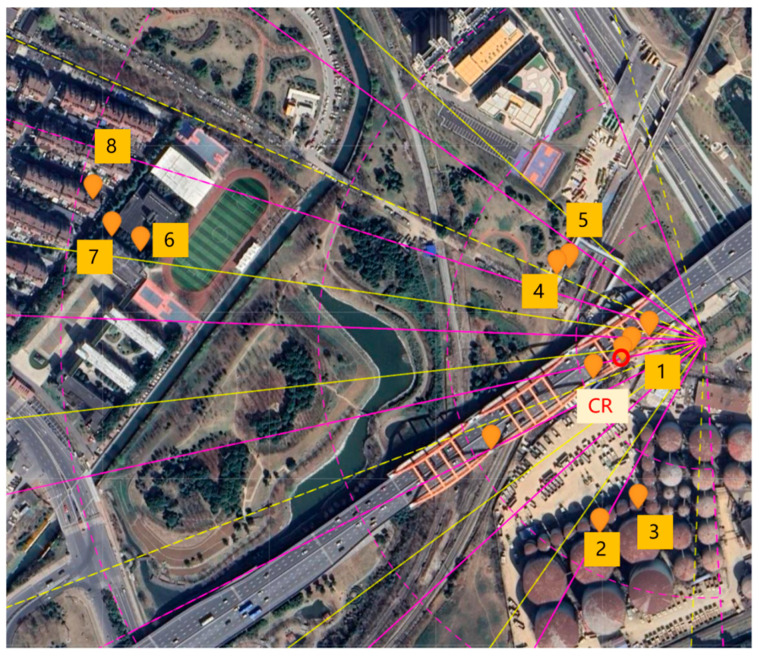
Setup for SHM of gNB mast (the numbers indicate the selected PSs).

Monitoring telecommunication masts is intrinsically important and can be considered an independent application of radar interferometry using a gNB base station. Currently, external sensors are typically required [35,45]; however, the proposed method allows estimation of the mast’s structural characteristics directly from gNB data. The experimental setup used for this application is shown in Figure 17. Eight PSs were selected, distributed over an azimuthal range of approximately 96°. These PSs were positioned closer to the gNB than those used for the bridge monitoring experiments to ensure a high SNR. The measurement session was carried out under natural excitation (e.g., wind) during a time interval without significant sway effects. The natural frequency spectrum and the corresponding oscillation axis were analysed. Any variation in these parameters may indicate potential structural changes in the mast.

**Figure 18:**
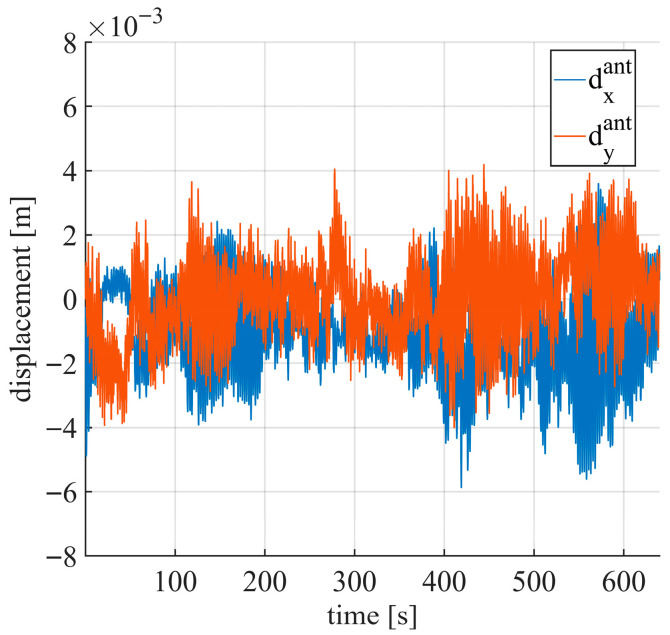
Movement of the mast retrieved using (10).

**Figure 19:**
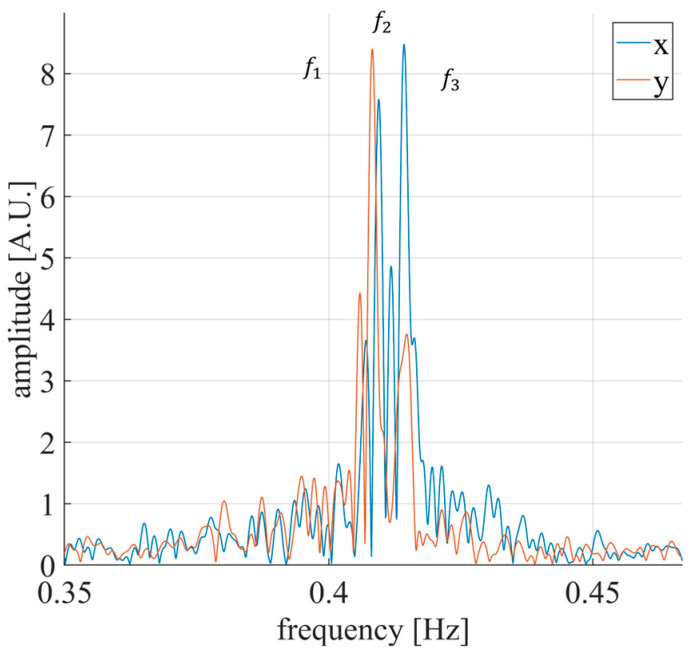
Natural frequency spectrum of the antenna mast.

The x and y components of the mast movement, retrieved using Equation (10), are shown in Figure 18. Mast displacements ranged from −6 mm to 4 mm. The natural frequency spectrum, presented in Figure 19, reveals three main frequencies: $f_{1}=0.408$ Hz, $f_{2}=0.409\;Hz$, and $f_{3}=0.414\;Hz$. These frequencies are consistent with those observed in Figure 15. It should be noted that the spectra in Figure 15 and Figure 16 are related to the projections of the mast movement on the radar–bridge line and do not represent the actual natural frequencies of the mast.

**Figure 20:**
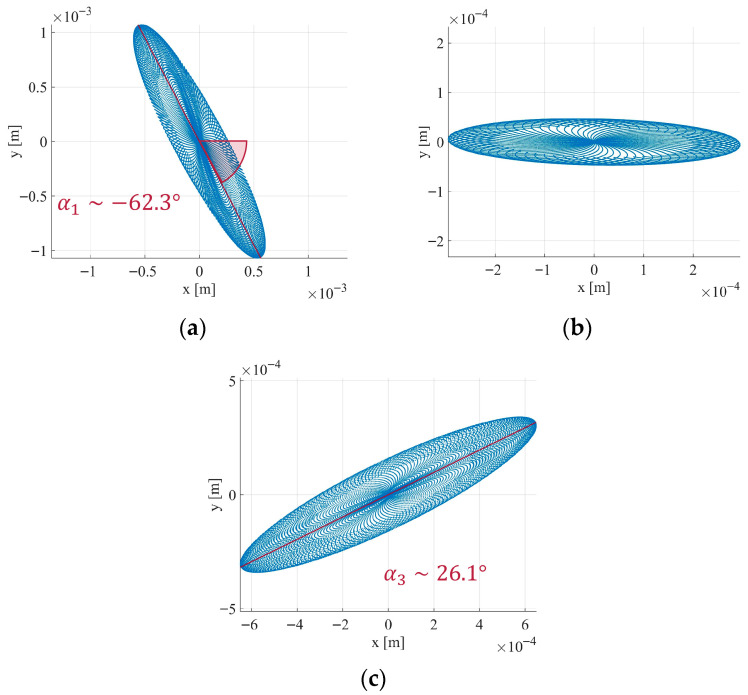
Natural axis related to: (**a**) $f_{1}$; (**b**) $f_{2}$; (**c**) $f_{3}$.

The oscillation axes associated with each natural frequency were determined by filtering the mast movement in Figure 18 around the respective frequencies. Three Butterworth band-pass filters with 20 poles were applied. The resulting scatterplots of displacement at each frequency are shown in Figure 20. The natural axis related to $f_{1}$ has an inclination of approximately $\alpha_{1}\approx -62.3{}^{\circ}$ (Figure 20a), for $f_{2}$ it is $\alpha_{2}\approx 0{}^{\circ}$ (Figure 20b), and for $f_{3}$ it is $\alpha_{3}\approx 26.1{}^{\circ}$ (Figure 20c).

## 4. Discussion

The proposed method was developed to estimate and compensate for antenna mast motion and atmospheric effects affecting the interferometric phase. It was validated by retrieving both quasi-static and dynamic displacements of the monitored structure, namely a bridge. Nevertheless, the performance of the method is subject to several constraints that should be carefully considered when extending its application to different measurement scenarios.

The method relies on the simultaneous estimation of the mast displacements along different directions using multiple stable targets (PSs). Accurate retrieval of the orthogonal components of mast displacement through multiparametric linear regression requires a sufficiently wide angular distribution of PSs, particularly in azimuth. In the scenario analysed in Section 3.1 and Section 3.2, the PSs span an azimuthal range of approximately 126° and an elevation range of 23.7°. The relatively limited angular separation in elevation represents a potential source of uncertainty, especially for the estimation of the vertical displacement component. However, this limitation is not critical for the investigated application, as mast motion predominantly occurs in the horizontal plane. Consequently, the vertical displacement component, although potentially affected by higher uncertainty, remains small and does not significantly impact the overall motion compensation. This limitation may become more relevant in applications where vertical displacements are non-negligible or when a more accurate three-dimensional motion reconstruction is required.

Regarding PSs identification, suitable targets can be selected based on a temporal analysis of the signal amplitude and may include scatterers of different nature, such as high reflective elements from buildings or other stable structures. A fundamental requirement is that these targets remain motionless over the observation interval. While individual PSs may exhibit independent vibrations modes, their impact is largely mitigated by the multiparametric regression approach. Conversely, coherent displacements affecting multiple PSs, such as those induced by thermal expansion or slow structural deformations, may introduce systematic biases in the estimated mast displacement. In such cases, additional calibration procedures or external measurements may be required.

Both quasi-static and dynamic measurements revealed a sway effect associated with thermal expansion [31,43] which can inhibit the monitoring of slow structural movements. Currently, this sway is usually detected using inertial sensors embedded in the antenna mast. Such sensors must be synchronized with the gNB time-stamp and further processed to retrieve antenna displacement or attitude information [31]. The proposed method enables the estimation of sway directly from the gNB radar data, allowing direct displacement retrieval without the need for auxiliary sensors, at the expense of requiring accurate knowledge of the spatial distribution of multiple PSs.

Another requirement for JCAS-based monitoring is the suppression of mast vibrations occurring at frequencies comparable to those of the monitored structure. The proposed method achieved a mast vibration attenuation of up to 27.5 dB (see Figure 16). For comparison, other approaches for radar applications employing external sensors report vibration suppression levels ranging between 3 dB and 25 dB [46,47]. Therefore, the proposed method provides comparable or superior vibration mitigation while avoiding the use of additional instrumentation. Moreover, the method significantly reduces noise associated with the sway effect (see Figure 15), which would otherwise hinder the correct identification of the natural frequency spectrum of the bridge under test.

Finally, the results obtained from the monitoring of the telecommunication mast (Section 3.3) confirm the effectiveness of the proposed method in isolating the structural dynamics of the mast, specifically in terms of its natural axes and frequencies. The analysis shows that a relatively small number of PSs (eight) is sufficient to estimate the orthogonal components of the mast displacement, provided that the angular separation between the PSs is sufficiently large (at least 60°). Using multiparametric regression, the phase contributions associated with the mast motion were successfully estimated, enabling a reliable retrieval of the actual gNB displacement.

The displacement analysis revealed a mast motion within ±6 mm under natural excitations, consistent with the expected quasi-static behavior of telecommunication pylons under low wind intensity. Furthermore, the retrieved natural frequencies ($f_{1}=0.408\;\mathrm{Hz}$, $f_{2}=0.409\;\mathrm{Hz}$, and $f_{3}=0.414\;\mathrm{Hz}$) are in agreement with characteristic oscillation frequencies reported for similar structures [35]. Such correspondence demonstrates that the gNB-based radar system can provide coherent interferometric measurements suitable for structural modal analysis. The identification of the vibration axes corresponding to each natural frequency reveals that the mast oscillates along three preferential directions.

The results presented in this study primarily demonstrate the operating principle of the proposed technique for mast health monitoring. While the method shows promising capabilities for tracking modal parameters, a comprehensive sensitivity analysis has not yet been conducted. Long-term deployments will therefore be required to fully characterize the influence of environmental factors, such as temperature variations and solar radiation, on the estimated natural frequencies and mode shapes, which is essential for reliable application in real-world structural health monitoring scenarios.

## 5. Conclusions

A multiparametric method has been proposed to estimate and compensate for atmospheric effects and gNB antenna motion in interferometric measurements for JCAS applications. The method is based on multiple linear regression based on the spatial distribution of PSs in the observed scene and has been specifically developed for gNB interferometric radar applications. The proposed approach has been experimentally validated through the retrieval of quasi-static and dynamic displacements of a monitored bridge. For quasi-static measurements, a controlled target was used to simulate slow structural movements, which were accurately recovered after disturbance compensation. For dynamic monitoring, the method demonstrated the capability to effectively suppress mast-induced vibrations, achieving an attenuation of approximately 27 dB.

Finally, the SHM of the telecommunication mast was successfully tested during a third measurement session conducted under negligible sway and limited atmospheric variability. The results demonstrated the ability of the proposed method to retrieve the modal and spectral characteristics of the mast, highlighting its potential for long-term monitoring of telecommunication infrastructures.

Future work will focus on extending the applicability and robustness of the proposed method to more general monitoring scenarios. In particular, further investigations will address configurations requiring accurate three-dimensional displacement reconstruction and the mitigation of potential biases arising from coherent motions of permanent scatterers, such as those induced by thermal effects or slow deformations. Moreover, dedicated long-term measurement campaigns will be conducted to assess the sensitivity, accuracy, and repeatability of the method under varying environmental conditions, which are essential for its reliable deployment in real-world structural health monitoring applications.

## Data Availability

No dataset is publicly available.

## Abbreviations

The following abbreviations are used in this manuscript:

gNB	next-generation Node B (5G base station)
JCAS	Joint Communication and Sensing
GBRI	Ground-Based Radar Interferometry
SHM	Structural Health Monitoring
SAR	Synthetic Aperture Radar
MIMO	Multiple Input Multiple Output
OFDM	Orthogonal Frequency Division Multiplexing
PS/PSs	Permanent Scatterer(s)
CR/CRs	Corner Reflector(s)
PRF	Pulse Repetition Frequency
OLS	Ordinary Least Squares
